# A Comprehensive Review: Robot-Assisted Treatments for Gait Rehabilitation in Stroke Patients

**DOI:** 10.3390/medicina60040620

**Published:** 2024-04-10

**Authors:** Yong-Hwa Park, Dae-Hwan Lee, Jung-Ho Lee

**Affiliations:** 1 Immanuel Medical Rehabilitation Hospital, 2140, Cheongnam-ro, Cheongju-si 28702, Republic of Korea; rmsid0245@naver.com (Y.-H.P.); dhlee8510@naver.com (D.-H.L.); 2 Department of Physical Therapy, University of Kyungdong, 815, Gyeonhwon-ro, Munmak-eup, Wonju-si 26495, Republic of Korea

**Keywords:** robot, stroke, gait, rehabilitation, physical therapy

## Abstract

Robot-assisted gait training (RAGT) is at the cutting edge of stroke rehabilitation, offering a groundbreaking method to improve motor recovery and enhance the quality of life for stroke survivors. This review investigates the effectiveness and application of various RAGT systems, including both end-effector and exoskeleton robots, in facilitating gait enhancements. The selection process for this comprehensive analysis involved a meticulous review of the literature from databases such as PubMed, the Cochrane Library, and EMBASE, focusing on studies published between 2018 and 2023. Ultimately, 27 studies met the criteria and were included in the final analysis. The focus of these studies was on the various RAGT systems and their role in promoting gait and balance improvements. The results of these studies conclusively show that patients experience significant positive effects from RAGT, and when combined with other physiotherapy methods, the outcomes are notably superior in enhancing functional ambulation and motor skills. This review emphasizes RAGT’s capability to deliver a more customized and effective rehabilitation experience, highlighting the importance of tailoring interventions to meet the specific needs of each patient.

## 1. Introduction

Strokes are a major global health issue and are one of the primary causes of long-term disability. Each year, strokes affect millions of individuals worldwide, resulting in a wide range of physical impairments. Among these, gait dysfunction and mobility impairment are particularly significant, profoundly impacting the quality of life and independence of stroke survivors [1]. The pathophysiology behind stroke-induced motor deficits is complex, involving neural damage that disrupts motor control and coordination, which are necessary for walking. This disruption manifests as various gait abnormalities, such as reduced walking speed, asymmetrical gait patterns, and decreased balance [2]. These abnormalities compromise functional mobility and significantly increase the risk of falls and related injuries. Addressing these gait dysfunctions is critical, as they limit the ability of stroke survivors to perform daily activities and reduce their overall quality of life. The comprehensive management of these impairments involves understanding the intricate neural and muscular changes that occur post-stroke and developing effective rehabilitation strategies to improve mobility and independence [3].

Traditional gait rehabilitation post-stroke primarily involves intensive physical therapy interventions that require significant human resources and time commitment. These traditional methods typically entail one-on-one sessions with physical therapists, where manual assistance plays a crucial role [4,5]. The techniques mainly focus on repetitive task-specific training aimed at improving walking ability, strength, and balance. While these methods can be effective, they are inherently limited by various factors. Firstly, maintaining the intensity and consistency required for optimal recovery can be challenging [4,5,6]. High-intensity, repetitive training is essential for harnessing the brain’s plasticity and promoting motor learning. However, the physical demands on both the patient and the therapist can hinder the necessary intensity and duration of training being achieved. Moreover, the availability of skilled therapists and the patient’s access to regular therapy sessions can be limited, especially in resource-constrained settings [6].

In recent years, the field of stroke rehabilitation has undergone a significant transformation with the emergence of robot-assisted therapy, marking a paradigm shift in addressing the limitations of traditional rehabilitation methods. Recognizing the need for more targeted, intensive, and engaging rehabilitation strategies, robot-assisted gait training (RAGT) has emerged as an innovative approach [7]. RAGT utilizes advanced robotic devices and systems meticulously engineered to support, enhance, and guide the lower limbs during walking [8,9,10]. These technologies range from wearable exoskeletons that closely align with the user’s body to end-effector devices like robotic treadmills, each offering unique mechanisms to assist with gait training. Exoskeletons provide direct physical support to the legs, actively assisting with leg movements to compensate for weaknesses and ensure proper gait patterns [9,10]. On the other hand, end-effector devices focus on guiding the feet along a predefined path, offering a different but equally effective approach to gait rehabilitation. Integrating RAGT with traditional physical therapy holds promise for improving outcomes post-stroke by addressing both the physical and neurological aspects of gait rehabilitation, thus offering a more comprehensive approach to stroke rehabilitation [8,9,10,11].

The fundamental objective of RAGT in the realm of stroke rehabilitation is to offer a training environment characterized by high intensity, repetitive practice, and task-specific exercises [9,10]. This approach is not arbitrary but is meticulously designed based on robust principles of rehabilitation science. High-intensity training is essential to challenge the motor system sufficiently, thereby facilitating significant improvements in muscle strength and endurance [12]. The repetitive nature of the training is critical for ingraining motor skills, a concept rooted in the principle of motor learning, which posits that the continuous practice of a skill enhances its execution. These robotic systems often incorporate sensors and software algorithms that allow for real-time feedback and adjustments. This capability enables the training to be highly personalized, adapting to the individual’s specific needs and progress. The interactive nature of RAGT, often augmented with virtual reality or gaming elements, enhances patient engagement and motivation, which are key factors in successful rehabilitation [12,13].

One of the significant advantages of RAGT over traditional gait rehabilitation methods is the ability to deliver consistent and controlled training [11,14]. The robotic devices can maintain precise control over movement patterns, speed, and resistance, ensuring that patients perform exercises with the correct form and intensity. This level of control is challenging to achieve with manual therapy alone. Moreover, RAGT allows for the delivery of intensive training without the physical strain on therapists and with reduced risk of injury to patients. This aspect is particularly important for patients with severe impairments who require substantial support during training [15].

The rationale for incorporating RAGT in stroke rehabilitation is deeply rooted in the scientific principles of neuroplasticity and motor learning [14,16]. Neuroplasticity refers to the brain’s remarkable ability to reorganize itself by forming new neural connections in response to learning or after injury. In the context of stroke rehabilitation, this plasticity is critical for the recovery of motor functions that are lost or impaired due to brain injury. RAGT leverages this principle by providing consistent and repetitive training, which is essential for stimulating and reinforcing these new neural pathways [16]. Such a repetitive practice of performing walking movements using robotic assistance helps to ‘retrain’ the brain, gradually restoring the neural circuits necessary for motor function. This is especially important in stroke rehabilitation, where the goal is to relearn and improve motor skills such as walking [17].

Another key aspect of RAGT is its capacity for personalization and adaptability to the individual needs of each patient [9,12]. Strokes affect individuals in diverse ways, leading to varying degrees and types of motor impairments. RAGT systems can be adjusted in terms of support, resistance, and pace, allowing therapists to tailor the rehabilitation program to the specific requirements and abilities of each patient [18]. This personalized approach ensures that patients are neither under-challenged nor overstrained, optimizing the rehabilitation process [12,19]. The interactive nature of RAGT is a significant advancement over traditional rehabilitation methods. These robotic systems often incorporate elements of virtual reality, gaming, and real-time feedback, making the rehabilitation process more engaging and motivating for patients [15,18]. This engagement is crucial as it can significantly enhance patient motivation and adherence to rehabilitation programs [19].

The primary objective of this review is to synthesize existing evidence on the effectiveness of robot-assisted gait training for improving gait and mobility in patients who have experienced a stroke [19]. By doing so, it aims to provide insights into the potential benefits, challenges, and future directions of RAGT in the context of stroke rehabilitation. This review seeks to inform clinicians, researchers, and healthcare policymakers, contributing to the optimization of stroke rehabilitation strategies and enhancing the quality of care for stroke survivors [20].

## 2. Materials and Methods

### 2.1. Data Sourse and Search Strategy

A thorough literature search was conducted using the databases PubMed and the Cochrane Library, selected for their comprehensive collections of medical and health-related research. The temporal scope of the search was focused on studies published in English from 2018 to 2023, to ensure inclusion of the most current and relevant data. The search utilized a combination of keywords: “stroke”, “robot”, and “gait”. These terms were specifically linked using the Boolean operator “AND” to ensure a comprehensive retrieval of studies relevant to the intersection of these key concepts. This strategic use of “AND” aimed to include studies that simultaneously addressed all three aspects: stroke, robot-assisted interventions, and gait analysis or rehabilitation.

In this study, the process of selecting and classifying relevant studies was rigorously conducted in accordance with the PRISMA (Preferred Reporting Items for Systematic Reviews and Meta-Analyses) guidelines. In line with the PRISMA framework, the initial stage of the study involved a database search to identify studies related to RAGT in stroke rehabilitation. During this stage, any duplicate papers and studies not written in English were meticulously excluded. In the screening phase, the titles and abstracts of the collected studies were reviewed against predefined inclusion and exclusion criteria.

At the eligibility stage, the full text of the remaining studies was thoroughly reviewed, and each study was carefully assessed to ensure relevance and compliance with the inclusion criteria. Finally, in the inclusion phase, studies that met all criteria were selected for detailed analysis and data extraction. From each study, information was extracted about study design, participant demographics, details of the RAGT intervention, comparison interventions, outcome measures, and main findings. To minimize bias and maximize the reliability and objectivity of the selection process, this entire procedure was independently conducted by three peer reviewers. Discrepancies encountered during the selection process were resolved through collaborative discussions or, when necessary, by consulting a fourth subject-matter expert.

### 2.2. Selection Criteria

#### 2.2.1. Study Types

In this review, the focus was placed on studies that explored the use and effectiveness of RAGT in the context of stroke rehabilitation. The research included was centered specifically on the application of RAGT in rehabilitation. Studies not directly related to robot-assisted gait training for stroke rehabilitation were excluded. Additionally, studies that focused on other forms of rehabilitation or robotic technology not directly related to gait training were also excluded.

#### 2.2.2. Participant Types

Patients diagnosed with stroke by medical professionals were included in the study regardless of the location and type of stroke onset. There were no restrictions on the patients’ age, gender, or nationality. Studies involving populations other than adult patients who experienced a stroke, such as those involving pediatric subjects or diseases unrelated to stroke, were also excluded.

#### 2.2.3. Intervention and Control Types

Studies that applied lower-limb rehabilitation using RAGT to the experimental group were selected, and robotic rehabilitation applied to the upper limbs was excluded. However, there were no specific standards for RAGT robot type, application method, and application period. The control group was set as a group that used existing treatments and general physical therapy and exercise therapy.

#### 2.2.4. Types of Outcome Measurements 

To measure the outcome of the study, studies that conducted evaluations related to lower extremity walking were selected, and papers with at least one of five measurements (FAC, BBS, TUG, 10MWT, 6MWT) were selected. Papers that only conducted other evaluations were excluded.

### 2.3. Quality Assessment

The assessment of randomized controlled trials (RCTs) was executed using version 5.1.0 of the Cochrane Risk of Bias tool. This methodology involves scrutinizing various critical factors to ascertain possible biases in the research. Key factors analyzed include the procedure for generating random sequences (aiming to make participant allocation unpredictable), the concealment of allocation (to prevent the influence on participant assignment by researchers), the blinding of both patients and staff (to minimize bias risks in the administration of treatments and evaluation of outcomes), the management of incomplete data outcomes (to guarantee that absent data do not skew the study’s findings), the prevention of selective outcome reporting (to ensure the inclusion of all anticipated results), and the identification of any additional sources of potential bias. For the sake of maintaining neutrality and precision in the quality evaluation, two independent researchers conducted the assessments. Whenever there was a discrepancy in their evaluations, they would revisit and meticulously review the contentious study. Should disagreements remain post-review, a resolution was sought through dialogue with an impartial third party, ensuring a unified conclusion.

## 3. Results

### 3.1. Literature Search

**Figure 1:**
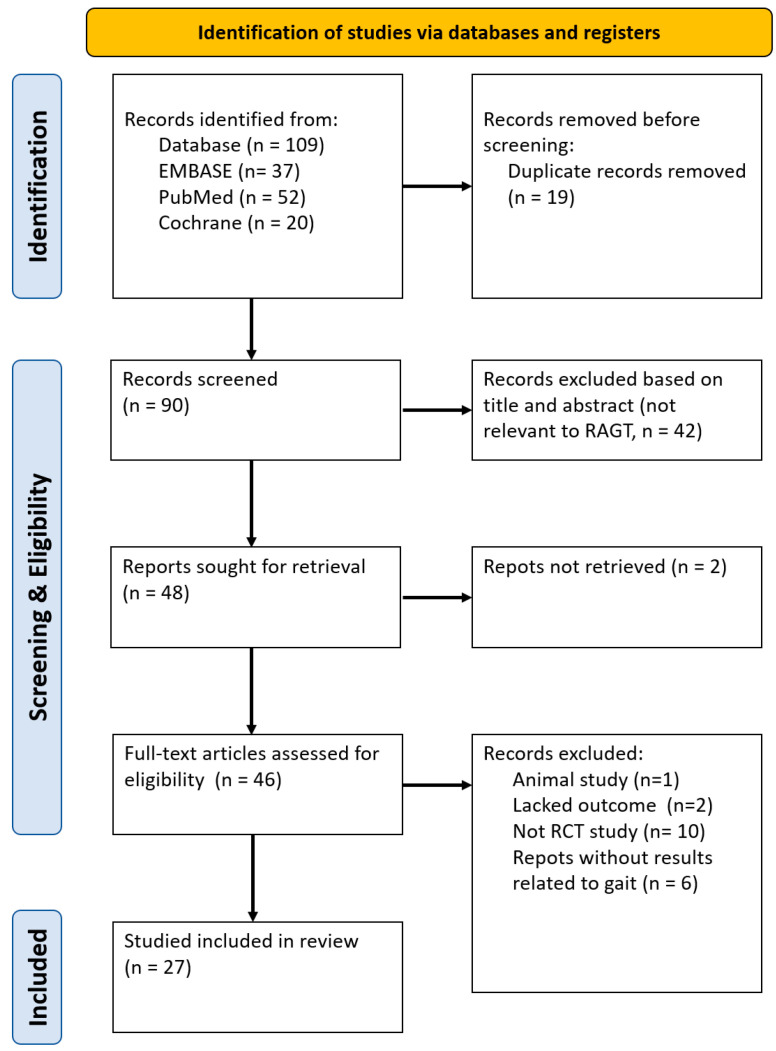
Flow diagram representing the study selection process.

Figure 1 shows a flow diagram of the study’s selection strategy. This paper selected studies related to lower limb robotic rehabilitation applied to patients following a stroke based on articles sourced from the EMBASE, PubMed, and Cochrane databases. Initially, a total of 37, 52, and 20 articles were identified from the EMBASE, PubMed, and Cochrane databases, respectively. Nineteen duplicate articles were excluded from the selection process. Upon reviewing the titles and abstracts, 42 articles were further excluded as they were not relevant to RAGT. The full texts of the remaining 46 articles were then evaluated for eligibility.

Three articles were excluded due to various reasons, including being animal studies and lacking outcome information. Among the selected studies, sixteen were excluded because they were non-RCTs (*n* = 10) or lacked assessments related to walking functions (*n* = 6) such as the Functional Ambulatory Category (FAC), 10-Meter Walk Test (10MWT), 6-Minute Walk Test (6MWT), Timed Up-and-Go test (TUG), Fugl–Meyer Assessment for Lower Extremity (FMA-LE), or Berg Balance Scale (BBS). Ultimately, 27 studies were included in this systematic review [21].

### 3.2. Study Characteristics

The systematic review encompasses a wide array of randomized controlled trials evaluating the efficacy of various RAGT devices in improving mobility outcomes for patients following a stroke. A total of 27 studies were included, employing diverse robotic systems such as the Morning Walk [22,23,24], Lokomat [25,26,27,28,29,30,31], G-EO System Evolution [16,31,32], and others, to facilitate gait rehabilitation. These studies vary in sample size, interventions, control conditions, and outcome assessments, providing a comprehensive overview of the current state of research in this field (Table 1).

All studies were RCTs, with sample sizes ranging from small (six participants per group in the study by Federica [33]) to relatively large (seventy-five participants per group in the study by Chang [10]). This variation in sample sizes reflects the exploratory nature of some studies and the more confirmatory approach of others. The interventions included a variety of robotic devices designed for gait training. Notably, devices such as the Lokomat and G-EO System were frequently used across studies, indicating their prominence in the field. Interventions often contrasted RAGT with conventional therapy approaches [34,35,36,37,38], such as physical therapy exercises, therapist-assisted gait training, or neurodevelopmental techniques. Control conditions varied significantly across the studies, ranging from conventional physical therapy and overground gait training to other forms of robotic gait training or biofeedback mechanisms. This diversity in control conditions underscores the broad spectrum of standard care practices and alternative rehabilitation strategies being investigated [27].

A wide range of outcome measures were utilized to assess the efficacy of RAGT, including the FAC, 10MWT, 6MWT, TUG, FMA-LE, and BBS [39,40,41,42,43,44,45], among others. These assessments cover various aspects of mobility, gait, balance, and functional independence, providing a multidimensional view of patient outcomes. The inclusion of studies with diverse robotic systems, control conditions, and outcome measures offers a broad perspective on the potential benefits and challenges associated with RAGT for stroke rehabilitation [24].

**Table 1:** Study Characteristics.

Study	Study Design	Sample Size (E/C)	Intervention		Assessment
			Experimental Group	Control Group	
Kim (2022) [22]	RCT	20/20	Morning walk-assisted gait training (biometric data control group)	Morning walk-assisted gait training (therapist control group)	FAC, 10MWT, TUG, BBS
Aprile (2022) [32]	RCT	19/17	G-EO system evolution (end-effector system gait/trunk group)	G-EO system evolution (end-effector system gait group)	FAC, 10MWT, 6MWT, TUG, BBS
Kim (2020) [16]	RCT	14/14	G-EO system evolution	30% body weight support and a speed of 0.8 km/h	FMA, 10MWT, TUG
Kim (2019) [25]	RCT	10/9	Lokomat (RAGT + CPT 4 weeks -> CPT 4 weeks)	Lokomat (CPT 4 weeks -> RAGT + CPT 4 weeks)	FAC, 10MWT, FMA-LE
Belas (2018) [26]	RCT	7/8	Lokomat + CPT	TAGT + CPT	BBS, TUG
Tamburella (2019) [27]	RCT	6/6	Lokomat + EMGB	Lokomat + Rb	FAC, BBS
Alingh (2021) [39]	RCT	17/15	AANmDOF Robotic (LOPESII)	CPT	10MWT, 6MWT, TUG, FMA-LE
Yu (2021) [40]	RCT	27/27	A3(NX) Gait Training and Evaluation system	Gait training	TUG, FMA
Zhang (2023) [41]	RCT	20/20	MANBUZHEKANGFU (GR-A1)	CPT	FAC, 6MWT, FMA-LE,
Lee (2022) [24]	RCT	33/10	Morning walk-assisted gait training (pelvic off *n* = 11, pelvic control *n* = 12, CIMT *n* = 10)	Treadmill + CPT	10MWT, TUG, BBS
Kang (2021) [42]	RCT	15/15	SUBAR	CPT	FAC, 10MWT, TUG, BBS
Talaty (2023) [28]	RCT	15/15	Lokomat + CPT	TAGT + CPT	FAC, 10MWT
Mustafaoglu (2020) [29]	RCT	34/17	Lokomat (group 1: RAGT + CPT *n* = 17, group 2: RAGT *n* = 17)	CPT	6MWT, FMA-LE
Meng (2022) [43]	RCT	128/61	Walkbot robotic (group 1: RAGT *n* = 62, group 2: RGAT + ELLT *n* = 66)	CPT	FAC, 6MWT, TUG
Miyagawa (2023) [44]	RCT	17/19	Curara + OT	CPT + OT	10MWT, 6MWT, BBS
Yokota (2023) [34]	RCT	12/10	Hybrid assistive limb + CPT	CPT	FAC
Bergqvist (2023) [35]	RCT	27/14	Hybrid assistive limb + CPT	CPT	FAC, 6MWT, 10MWT, BBS
Yeung (2021) [36]	RCT	30/17	Dynamixel MX-106R PAAR (power-assisted ankle robot + CT *n* = 14, swing-controlled ankle robot + CT *n* = 16)	CT	10MWT, BBS
Palmcrantz (2021) [45]	RCT	13/28	Hybrid Assistive Limb	No specific training intervention	10MWT, 6MWT, FMA, BBS
Chang (2023) [10]	RCT	75/75	Angel Legs M20 + Gait training	Gait training	FAC, 10MWT, 6MWT, FMA-LE, BBS
Louie (2020) [37]	RCT	20/20	EksoGT powered robotic exoskeleton + CPT	CPT	6MWT, BBS,
Wright (2021) [38]	RCT	16/18	AlterG Bionic Leg orthosis + CPT	CPT	FAC, 6MWT, TUG, BBS, DGI
Lee (2023) [23]	RCT	26/23	Morning walk + CPT	CT	10MWT, FMA-LE, BBS
Choi (2022) [30]	RCT	18/6	Lokomat PRO + NDT (BWS 30% *n* = 6, 50% *n* = 6, 70% *n* = 6)	Treadmill + NDT	10MWT, TUG, BBS
Seo (2018) [33]	RCT	6/6	Walkbot + AAN (unaffected limb)/ FA (affected limb)	Walkbot + FA (unaffected limb)/AAN (affected limb)	FAC, FMA-LE
Kayabinar (2021) [20]	RCT	15/15	VR + RoboGait (Exoskeleton)	RoboGait (Exoskeleton)	FAC, 10MWT, BBS
Pournajaf (2022) [31]	RCT	30/59	End-effector (G-EO) + + Overground gait training	Exoskeleton (Lokomat) + Overground gait training	10MWT, 6MWT, TUG

RCT, randomized controlled trial; E, experimental group; C, control group; FAC, functional ambulation category; 10MWT, 10 m walk test; 6MWT, 6-min walk test; TUG, timed up-and-go test; FMA-LE, Fugl–Meyer assessment lower extremity; BBS, Berg balance scale; DGI, dynamic gait index; RGAT, robotic-assisted gait training; CPT, conventional physical therapy; TAGT, therapist assist gait training; EMGB, electromyographic based biofeedback; NDT, neurodevelopmental techniques; CIMT, constraint-induced movement therapy; CT, conventional training; ELLT, enhanced lower limb therapy; OT, occupation therapy; BWS, body weight support; VR, virtual reality, AAN, assist-as-needed; FA, fully assisted mode.

### 3.3. Types of Robots Used in Treatment

Based on Table 2’s comprehensive overview of research characteristics by robot type, we can derive critical insights into the effectiveness and application of RAGT across various studies. This analysis highlights the differences in outcomes related to end-effector robots [19,22,23,32], fixed exoskeletons [20,24,25,26,27,28,29,30,33,39,40,41,42,43], wearable exoskeletons [34,35,36,38,44,45], and the combination of end-effector and fixed exoskeleton robots in the rehabilitation of individuals with mobility impairments [31].

Studies involving end-effector robots show significant improvements in functional ambulation categories, balance [22,32], muscle strength [32], and walking tests among participants. For instance, the use of the end-effector RAGT for 30 min over 4 weeks resulted in significant improvements across all outcome measures, with notable progress in the FAC [23]. However, not all studies reported differences between the intervention and control groups, indicating the necessity of further research to identify the conditions under which these robots are most effective. Fixed exoskeletons, such as the G-EO system evolution [16,31,32] and Lokomat [25,31], facilitated increased cortical activation and significant improvements in motor function scores (FMA) [22,29,40]. The interventions, typically spanning 4 to 6 weeks, demonstrated varying degrees of effectiveness in improving gait parameters, balance, and functional independence. Some studies highlighted the added value of combining robotic gait training with conventional physiotherapy techniques to achieve better outcomes [25,26,36,38].

Wearable exoskeletons, including devices like the HAL [34,35,45] and the Curara system [44], showed that, while significant intragroup improvements in gait speed, stride length, and cadence were observed, comparisons between groups often did not reveal significant differences. This suggests that, while wearable exoskeletons can enhance specific aspects of gait within an individual [36,38], their superiority over other forms of rehabilitation is not always clear [34]. The study involving the combination of end-effector and fixed exoskeleton robots indicated significant benefits in walking speed, endurance, balance, and the performance of activities of daily living (ADL) [31]. This approach suggests that integrating different types of robotic technologies could potentially offer a more comprehensive rehabilitation strategy, accommodating a wider range of impairments.

When comparing the effects of RAGT and traditional walking training over 4 weeks and 6 weeks, a significant difference in walking ability was observed between the two groups [16,22,23,24,25,26,27,28,30,40,41,42]. However, interestingly, as the duration of training increased, the difference between the groups diminished considerably [26]. This suggests that, over a longer training period, both groups achieved a similar level of improvement in walking ability [22,23,24,25,26]. Therefore, the effectiveness of walking training may be associated with the duration of training, indicating that robotic training and traditional training can achieve comparable results over time [23,24,25,26,27,28].

### 3.4. Quality Assessment

**Figure 2:**
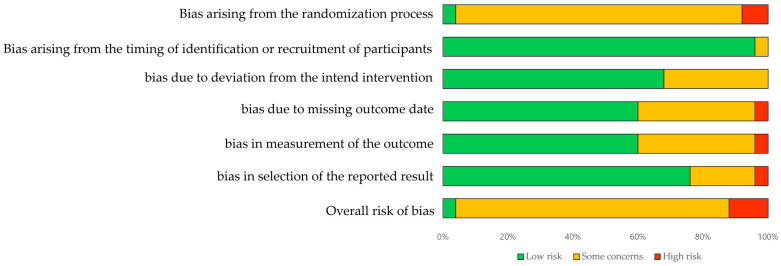
Risk of Bias 2.0 summary: authors judgements for each risk of bias domain ac included studies.

The risk of bias encompasses systematic errors or flaws in the design, execution, or analysis of a research study, impacting the validity and reliability of its findings (Figure 2, Table 3). It is crucial to identify and assess potential biases to accurately interpret study results and make evidence-based decisions [46,47]. The major types of bias include selection bias, performance bias, detection bias, attrition bias, and reporting bias, which must be evaluated and mitigated through considerations of study design, methodological quality, data collection and analysis methods, and conflicts of interest. Biased study results can lead to an overestimation or underestimation of treatment effects, incorrect conclusions, or misguided policy decisions [47,48]. Therefore, enhancing the quality and credibility of research requires transparent and rigorous reporting and review processes to minimize bias and ensure robust findings.

D1a evaluates whether participants were blinded and randomized before enrollment, as prior knowledge of group assignment could affect study outcomes. Among the 25 studies, only one study blinded the participants, and this study compared differences between robot therapies. This indicates the impracticality of blinding when comparing robot therapy to traditional physiotherapy, given the specialized equipment involved [31]. Additionally, another two studies did not report the use of participant blinding, while it was not mentioned in the remaining studies [22,42,45]. D1b assesses whether the participants were recruited before randomization, as prior knowledge could influence outcomes. Among the 24 studies with low risk, most prevented participants from acquiring knowledge related to the study. One study was evaluated as having some concerns due to a lack of records regarding some patients’ concerns [44]. D2 evaluates the blinding of participants and therapists, as their awareness could lead to modified treatments, affecting outcomes. Among the 17 studies evaluated with low risk, one study lacked therapist awareness, and eight studies were rated with some concerns as therapists were aware but treatment modification was not feasible [16,22,24,39,41,43]. D3 assesses the role of blinding in outcome assessment. Fifteen studies were rated as low risk, indicating the blinding of assessors. Nine studies were rated with some concerns as assessors were not blinded, but dropout rates were minimal, thus posing a low risk of bias [22,39,40,41]. However, two studies had a high dropout rate (50%) [31,33], raising concerns about outcome distortion, leading to a high-risk rating. D4 evaluates the risk of outcome data distortion. Fifteen studies were evaluated as being low risk, indicating that appropriate measurement methods were used consistently across groups [31]. However, nine studies were rated with some concerns as assessors were aware of treatments, conducted by therapy experts, leading to a moderate risk. D5 assesses the significance of results, with 19 studies being rated as low risk.

## 4. Discussion

Robot-assisted gait training stands as an innovative approach in the rehabilitation of stroke survivors, offering a modern solution to improve walking function and enhance quality of life for those impacted by stroke-related mobility issues [49]. Utilizing state-of-the-art robotic devices, RAGT provides a structured platform for patients to engage in repetitive, task-specific, and interactive exercises, which are crucial for stimulating neuroplasticity and advancing functional recovery [40]. This method of rehabilitation is noted for its precision, adaptability, and the ability to deliver intensive, customized therapy that traditional rehabilitation methods may not fully achieve [33]. This discussion aims to reevaluate the key outcomes from recent research, assess the impact of robotic rehabilitation on lower extremity function, consider the most effective clinical applications, and identify limitations while proposing directions for future research.

### 4.1. Main Findings

The exploration into the efficacy of various robotic systems, including end-effector robots, fixed and wearable exoskeletons, and their combinations, has opened new avenues for targeted gait and balance rehabilitation. Each type of robot brings distinct benefits to the rehabilitation landscape [25,42,44].

End-effector robots, which directly interact with the patient’s limbs, have been instrumental in significantly enhancing functional ambulation and balance among stroke survivors [23,32]. Their design allows for a wide range of motion, making them particularly effective in simulating walking patterns that improve gait speed and stability, thereby fostering a more natural walking experience [22]. Fixed exoskeletons offer robust support and stability through their stationary setup, enabling intensive gait training sessions for stroke survivors [16,27]. The mechanical assistance offered by these exoskeletons is linked with heightened cortical activation, suggesting their role in not only augmenting physical capabilities but also in promoting neuroplasticity and motor function recovery [43]. This highlights the potential for fixed exoskeletons to contribute to the re-establishment of the neural pathways critical for gait and mobility.

Wearable exoskeletons represent a new phase in RAGT, merging mobility enhancement with rehabilitation efforts [34,44]. These devices support the body’s natural movements during walking, offering the promise of continuous, real-world practice [20,44]. Although they have demonstrated significant improvements in gait parameters within groups of patients who have experienced strokes, the evidence comparing their effectiveness to traditional rehabilitation methods varies [23,25,26,28]. This variability underscores the necessity for further comparative studies to solidify their role in stroke rehabilitation [23,25,26,28,30]. The combination of RAGT with conventional physiotherapy approaches has been a significant area of investigation, revealing a synergistic effect that appears to maximize patient outcomes [20,26,38,41]. This collaborative approach to rehabilitation leverages the strengths of both robotic technologies and traditional therapy methods, fostering an environment in which motor recovery and functional independence can be significantly enhanced [29,30,35]. The integration of RAGT into conventional therapy routines not only diversifies the rehabilitation regimen but also introduces varied stimuli that are crucial for neuroplasticity, thereby potentially accelerating recovery timelines and improving the quality of life of stroke survivors [30].

The advent of wearable exoskeletons has introduced a new dimension to RAGT, offering stroke survivors the possibility of enhanced mobility and independence [36]. These devices, designed to be used both within clinical settings and in the community, represent a significant step forward in making continuous, real-life rehabilitation feasible. However, despite the clear intragroup improvements observed with the use of wearable exoskeletons in gait parameters, their outright superiority over conventional rehabilitation methods is not uniformly acknowledged across the literature [34,35,44]. This discrepancy points to an urgent need for comprehensive, well-designed comparative studies that could provide clearer insights into the benefits of wearable exoskeletons relative to traditional therapies, considering various metrics of success including patient satisfaction, long-term outcomes, and cost effectiveness.

### 4.2. Impact of Lower Extremity Training & Optimal Clinical Application

Lower extremity training through RAGT has been demonstrated to have a profound impact on the rehabilitation outcomes of stroke survivors [16,43]. The integration of robotic devices in lower limb rehabilitation offers precise, consistent, and repetitive training sessions, which are crucial for neural reorganization and muscle re-education [23,28,29]. Studies have consistently shown that patients undergoing RAGT experience significant improvements in gait speed, balance, and overall walking ability compared to those receiving traditional therapy alone [16,23,36,38,43,45]. This improvement is attributed to the high dosage and intensity of task-specific exercises provided by robotic devices, which facilitate motor learning and contribute to the restoration of functional ambulation [20,30]. Moreover, RAGT has been shown to positively affect the psychological well-being of patients by enhancing their motivation and engagement during therapy sessions. The interactive nature of robotic devices, coupled with real-time feedback, creates a stimulating environment that encourages patients to achieve their rehabilitation goals. This psychological boost is essential, as it directly influences the patient’s commitment to the rehabilitation process and can lead to better outcomes [27,30].

For an optimal clinical application of RAGT in stroke rehabilitation, a comprehensive and individualized approach that integrates the latest robotic technologies with traditional rehabilitation practices is essential [23,30,38]. Effective application of RAGT requires tailoring interventions to match the unique needs of each patient, considering their specific impairments, recovery goals, and overall health status [23,25]. Personalization of the RAGT program is paramount. Assessing each patient’s motor, cognitive, and sensory abilities allows therapists to adjust the parameters of the robotic system, such as resistance, speed, and movement patterns, to suit the patient’s current capabilities and appropriately challenge them as they progress [32,33,40]. This level of customization supports the principles of neuroplasticity by encouraging the brain’s ability to reorganize and form new neural connections, thereby enhancing motor learning and recovery [20,31,33].

Moreover, integrating RAGT with sensorimotor training techniques can significantly augment rehabilitation outcomes [30,40,43]. By combining robotic gait training with exercises designed to improve sensory feedback, proprioception, and motor control, patients can experience a more comprehensive recovery, ultimately leading to better functional mobility and independence. Patient engagement and motivation are crucial factors in the success of RAGT [8,16,25]. Incorporating elements of gamification and virtual reality into the training sessions can make the rehabilitation process more enjoyable and engaging for patients, increasing their adherence to the therapy program. Furthermore, setting achievable goals and providing immediate feedback on progress can boost patient morale and encourage continued effort towards recovery [20,29,30,38].

Preparing patients for the transition back to community living is an essential component of RAGT. This involves training on a variety of surfaces, navigating obstacles, and simulating real-life situations to ensure that patients can apply the skills learned in therapy to their daily lives [20,30,34]. Emphasizing functional mobility and the ability to perform everyday activities is key to improving patients’ quality of life post-stroke. Lastly, long-term support and follow-up care are integral to sustaining the gains made during the rehabilitation process [28,34,45]. Providing patients with home-based exercises, periodic reassessments, and access to community resources can help maintain their progress and prevent regression. Continuous support not only aids in physical recovery but also addresses the ongoing emotional and psychological needs of stroke survivors [30,34,45].

In conclusion, the optimal clinical application of RAGT requires a patient-centered, interdisciplinary approach that leverages the capabilities of robotic technologies while incorporating traditional rehabilitation principles. By adopting a holistic, personalized, and progressive rehabilitation strategy, healthcare professionals can maximize the therapeutic potential of RAGT, leading to enhanced recovery outcomes for patients following a stroke.

### 4.3. Limitations and Suggestions for Further Studies

The field of RAGT for stroke rehabilitation, while promising, faces several limitations that highlight areas for future research and improvement [30,31,43]. A notable concern is the generalizability of study results, as many investigations involve small, homogenous participant groups, making it difficult to apply findings broadly across the diverse population of stroke survivors. Additionally, the long-term effectiveness of RAGT remains largely unexplored, with a need for more research to understand the persistence of benefits over time [31,32,39]. Comparative effectiveness research is also lacking, with few studies directly comparing different robotic devices or contrasting RAGT with traditional rehabilitation methods [16,25,31,40]. This gap in the literature makes it challenging for clinicians to make informed decisions about the best approaches for their patients [23,37,38]. Moreover, the high costs associated with robotic technology pose significant barriers to access and widespread implementation, underscoring the necessity for comprehensive cost–benefit analyses to justify the investment in RAGT.

The similarity in effectiveness between RAGT and conventional gait training, with increasing convergence as the duration of training extends, can be attributed to several factors [16,17,25]. Both methods target similar underlying mechanisms involved in gait rehabilitation, such as muscle strength, coordination, balance, and proprioception. Despite differences in the delivery of training (robotic assistance versus manual assistance), the fundamental principles of motor learning and neuroplasticity remain similar across both approaches [26,27,28,33]. Longer training periods provide ample time for individuals to practice and reinforce newly acquired motor patterns and skills. Additionally, individual variability in response to training may play a role. Some participants may respond more favorably to RAGT, while others may benefit more from traditional training methods [22,23,24,25,31]. However, as the training duration increases, the cumulative effects of training tend to outweigh individual differences, leading to converging outcomes between the two groups [21,22,23,24,25,26,27,41,43].

Addressing the limitations of RAGT research requires future studies to focus on engaging larger and more diverse sample sizes, which will significantly improve the generalizability of outcomes and ensure that RAGT’s advantages are accessible to a wider array of stroke survivors. Furthermore, the implementation of long-term follow-up studies is crucial for evaluating the enduring effects of RAGT on patient recovery trajectories, providing valuable insights into the long-lasting impact of such interventions [33,34,39,45]. Equally important is the need for direct comparative studies to evaluate the efficacy of various robotic systems against each other and compared to conventional rehabilitation methods [15,16,23,38]. This will enable the development of evidence-based guidelines that can inform clinical decision-making, ensuring that patients receive the most effective rehabilitation strategies tailored to their specific needs. Lastly, there is a compelling need to explore the synergistic potential of integrating RAGT with other therapeutic modalities, such as virtual reality and neuromuscular electrical stimulation [16,17,33,41,43]. Such multidisciplinary approaches could offer holistic rehabilitation solutions that optimize recovery outcomes by engaging patients in a more immersive and motivating training experience.

## 5. Conclusions

This systematic review thoroughly assessed the impact of RAGT on stroke rehabilitation, underscoring its significant potential to augment recovery outcomes. Our findings reveal that RAGT, through its various implementations like end-effectors and exoskeletons, acts as a valuable complement to traditional rehabilitation techniques. It notably enhances gait and balance, highlighting the effectiveness of combining RAGT with general therapies to achieve superior improvements in motor functions. There is a call for studies to explore long-term effects of RAGT to fully understand its benefits and to ensure that RAGT protocols are effectively integrated into clinical practices.

## Figures and Tables

**Table 2:** Research characteristics according to type of robot.

Robot Type	Study	Applied Robot	Intervention Periods	Assessment Period	Outcomes
End-effector	Lee (2023) [23]	End-effector RAGT	4 weeks	BF, AF	Significant improvements in all outcome measures; robot group improved more in FAC.
	Kim (2022) [22]	Morning walk	6 weeks	BF, AF	Significant improvements in FAC, MBI, BBS, TUG, 10MWT in both groups; no significant differences between groups.
	Aprile (2022) [32]	End-effector system	1 month	BF, AF	Improvement in balance ability in both groups; significant improvements in lower limb muscle strength and muscle tone in GTG group.
	Kim (2020) [16]	G-EO system evolution	4 weeks	BF, AF	Increased activation in primary sensorimotor cortex, supplementary motor area, premotor cortex; significantly better FMA scores in E RAGT group.
Exoskeleton (fix)	Kim (2019) [25]	Lokomat^®^ PRO	4 weeks	BF, AF	Significant differences in outcomes between groups; significantly greater improvements in FMA-LE and SARA in RAGT + CPT group.
	Belas (2018) [26]	Lokomat^®^ 5.0	5 months	BF, AF	Statistically significant improvements in balance, functional independence, and general ataxia symptoms in both groups; no significant between-group differences.
	Tamburella (2019) [27]	Lokomat	4 weeks	BF, AF	Significant improvements in gait/daily living activity independence and trunk control; EMGb more effective in reducing spasticity and improving muscle force.
	Alingh (2021) [39]	AANmDOF Robotic (LOPESII)	6 weeks	BF, AF, FU	Improvements in gait parameters and functional gait tasks; no significant group differences except for paretic knee flexion improvement in AANmDOF group.
	Seo (2018) [33]	Walkbot	10 weeks	BF, AF, FU	Clinical measurements improved in both groups; significant improvements in step length asymmetry ratio and hip maximal extension moment in group 1, and dorsiflexion angle in group 2.
	Yu (2021) [40]	G-EO system evolution	14 consecutive days	BF, AF, FU	Significant effect on changes in space parameters and FMA scores in RT group; no significant differences between groups.
	Zhang (2023) [41]	MANBUZHEKANGFU (GR-A1)	4 weeks	BF, AF	Experimental group significantly outperformed control group in various measures; significant improvement in co-contraction index of the knee in experimental group.
	Choi (2022) [30]	Lokomat^®^ PRO	6 weeks	BF, AF	Robot groups showed significantly better 10MWT results and shorter TUG than non-robot group; significant improvement in BBS scores for robot group A.
	Lee (2022) [24]	Morning walk	4 weeks	BF, AF	Significant improvements in BBS, TUG, MI-Lower in pelvic off group; greater improvement in TUG and BBS in pelvic on group, and in 10MWT and MI-Lower in CIMT group.
	Kang (2021) [42]	SUBAR	3 weeks	BF, AF	Significant improvements in MAS and step length in SUBAR group; control group showed significant improvements in BBS, MAS, and stride length.
	Talaty (2023) [28]	Lokomat	3 weeks	BF, AF, FU	Both groups showed significant improvements in several measures. CGT group had 45% more supplemental sessions than the Lokomat group. Both groups showed greater FIM improvement scores than a reference group with no supplemental therapy.
	Mustafaoglu (2020) [29]	Lokomat	6 weeks	BF, AF	Significant improvements in BI, 6MWT, SS-QOL, and SCT for primary outcomes and FMA-LE, CWT, RPE for secondary outcomes, except FWT. Group 1 showed significant improvement compared to group 2 and 3.
	Kayabinar (2021) [20]	RoboGait	6 weeks	BF, AF	Increase in single and dual-task gait speeds and cognitive dual-task performance in the study group. No significant difference between groups in all assessments after treatment.
	Meng (2022) [43]	Walkbot	4 weeks	BF, AF	Significant improvements in 6MWT, FAC, TUG, DTW, Tinetti’s test, BI, SS-QOL, and gait. RAGT group performed better in several measures compared to ELLT and CRT groups.
Exoskeleton (wearable)	Miyagawa (2023) [44]	Curara	15 days	BF, AF, FU	No significant difference in main outcomes between groups at the end of gait training. Significant intragroup improvements in gait speed, stride length, stride duration, and cadence.
	Yokota (2023) [34]	Hybrid assistive limb	20 sessions (5~6 day)	BF, AF, FU	No significant differences in primary outcomes. Apathy scale showed a decreasing trend in HAL group and a slight increasing trend in CPT group.
	Bergqvist (2023) [35]	Hybrid assistive limb	6 weeks	BF, AF, FU	No significant associations between MoCA Vis/Ex and 6MWT in robotic gait training group.
	Yeung (2021) [36]	Exoskeleton ankle robot (PAAR, SCAR)	20 sessions	BF, AF	Statistically significant improvements in functional ambulatory category and walking speed for SCAR and PAAR, respectively.
	Palmcrantz (2021) [45]	Hybrid assistive limb	6 weeks	BF, AF, FU	HAL group walked twice as far as conventional group during intervention. Post-intervention, only the conventional group showed significant improvement compared group.
	Wright (2021) [38]	AlterG Bionic Leg	10 weeks	BF, AF	Significant increases in walking distance, FAC, DGI, and BBS for over-ground robotic-assisted gait training. Improvements maintained at 22 weeks.
Exoskeleton(fix) and End-effector	Pournajaf (2022) [31]	G-EO (End-effector)	20 sessions	BF, AF	Robotic Group showed significant benefits in 10 MWT, 6 MWT, TUG, and MBI. Robot in gait speed, endurance, balance, and ADL. RobotEND-group improved walking speed more than RobotEXO-group.

BF, before test; AF, after test; FU, follow up test; FAC, functional ambulation category; MBI, modified Barthel index; BBS, berg’s balance scale, TUG, timed up and go test; 10MWT, 10 m walk test; GTG, gait trunk group; FMA, Fugl–Meyer assessment; FMA-LE, lower extremity; E-RAGT, end-effector robot-assisted gait training; SARA, scale for the assessment and rating of ataxia; CPT, conventional physical therapy; EMGb, electromyographic-based biofeedback; baPWV, brachial-ankle pulse wave velocity; RT, rehabilitation therapy; MI-Lower, motricity index of the lower extremities; CIMT, constraint-induced movement therapy, MAS, modified Ashworth scale, CGT, conventional gait training; FIM, functional independence measure, BI, Barthel index; 6-MWT, 6-min walk test; SS-QOL, stroke specific quality of life; SCT, stair climbing test; CWT, comfortable 10-m walk test; RPE, rate of perceived exertion; FWT, fast 10-m walk test; DTW, dual-task walking; ELLT, enhanced lower limb therapy, CRT, conventional rehabilitation; MoCA Vis/Ex, Montreal cognitive assessment; DGI, dynamic gait index.

**Table 3:** Methodological quality of included studies according to the tool Risk of Bias 2.0.

Study	D1a	D1b	D2	D3	D4	D5	Overall	Study	D1a	D1b	D2	D3	D4	D5	Overall
Kim (2022) [22]	H	L	Sc	Sc	Sc	Sc	Sc	Meng (2022) [43]	Sc	L	Sc	L	Sc	L	Sc
Aprile (2022) [31]	Sc	L	L	L	L	L	Sc	Miyagawa (2023) [44]	Sc	Sc	L	Sc	Sc	Sc	Sc
Kim (2020) [16]	Sc	L	Sc	L	L	Sc	Sc	Yokota (2023) [34]	Sc	L	L	Sc	Sc	Sc	Sc
Kim (2019) [25]	Sc	L	L	L	L	L	Sc	Bergqvist (2023) [35]	Sc	L	Sc	Sc	L	L	Sc
Belas (2018) [26]	Sc	L	L	L	L	L	Sc	Yeung (2021) [36]	Sc	L	L	L	L	L	Sc
Tamburella (2019) [27]	Sc	L	L	L	Sc	L	Sc	Palmcrantz (2021) [45]	L	L	L	L	L	L	L
Alingh (2021) [39]	Sc	L	L	Sc	L	L	Sc	Wright (2021) [38]	Sc	L	L	L	L	L	Sc
Yu (2021) [40]	Sc	L	L	Sc	L	L	Sc	Lee (2023) [23]	Sc	L	Sc	Sc	L	L	Sc
Zhang (2023) [41]	Sc	L	Sc	Sc	Sc	Sc	Sc	Seo (2018) [33]	Sc	L	L	H	L	H	H
Lee (2022) [24]	Sc	L	Sc	L	Sc	L	Sc	Choi (2022) [30]	Sc	L	L	L	Sc	L	Sc
Kang (2021) [42]	H	L	Sc	L	Sc	L	H	Kayabinar (2021) [20]	Sc	L	L	Sc	L	L	Sc
Talaty (2023) [28]	Sc	L	L	L	L	L	Sc	Pournajaf (2023) [31]	Sc	L	L	H	H	L	H
Mustafaoglu (2020) [29]	Sc	L	L	L	L	L	Sc								

D1a, risk of bias arising from the randomization process; D1b, risk of bias arising from the timing of identification or recruitment of participants in a randomized control; D2, risk of bias due to deviation from the intend intervention; D3, risk of bias due to missing outcome date; D4, risk of bias in measurement of the outcome; D5, risk of bias in selection of the reported result.
